# Exopolysaccharides Isolated from Milk Fermented with Lactic Acid Bacteria Prevent Ultraviolet-Induced Skin Damage in Hairless Mice

**DOI:** 10.3390/ijms18010146

**Published:** 2017-01-13

**Authors:** Masashi Morifuji, Masami Kitade, Tomoyuki Fukasawa, Taketo Yamaji, Masamitsu Ichihashi

**Affiliations:** 1 Food Science Research Labs, Meiji Co., Ltd., 540 Naruda, Odawara-shi, Kanagawa 250-0862, Japan; masami.kitade@meiji.com (M.K.); tomoyuki.fukasawa@meiji.com (T.F.); taketo.yamaji@meiji.com (T.Y.); 2 Saisei Mirai Clinic Kobe, Kobe Commerce, Industry and Trade Center Building 23F, 5-1-14 Hamabedori, Chuo-ku, Kobe-shi, Hyogo 651-0083, Japan; mm_ichihashi@hotmail.com

**Keywords:** fermented milk, exopolysaccharide, ultraviolet, skin damage, DNA damage

## Abstract

Background: We studied the mechanism by which fermented milk ameliorates UV-B-induced skin damage and determined the active components in milk fermented with lactic acid bacteria by evaluating erythema formation, dryness, epidermal proliferation, DNA damage and cytokine mRNA levels in hairless mice exposed to acute UV-B irradiation. Methods: Nine week-old hairless mice were given fermented milk (1.3 g/kg BW/day) or exopolysaccharide (EPS) concentrate (70 mg/kg BW/day) orally for ten days. Seven days after fermented milk or EPS administration began, the dorsal skin of the mice was exposed to a single dose of UV-B (20 mJ/cm^2^). Results: Ingestion of either fermented milk or EPS significantly attenuated UV-B-induced erythema formation, dryness and epidermal proliferation in mouse skin. Both fermented milk and EPS were associated with a significant decrease in cyclobutane pyrimidine dimers and upregulated mRNA levels of xeroderma pigmentosum complementation group A (XPA), which is involved in DNA repair. Furthermore, administration of either fermented milk or EPS significantly suppressed increases in the ratio of interleukin (IL)-10/IL-12a and IL-10/interferon-gamma mRNA levels. Conclusion: Together, these results indicate that EPS isolated from milk fermented with lactic acid bacteria enhanced DNA repair mechanisms and modulated skin immunity to protect skin against UV damage.

## 1. Introduction

Skin provides an effective barrier between an organism and its external environment that helps to reduce the risk of physical, chemical and microbial damage. Exposure to ultraviolet (UV) radiation has deleterious effects on skin, including sunburn, immune suppression, dryness, wrinkling, mottled pigmentation and skin cancer [1]. A variety of dietary supplements is known to have beneficial effects on skin health. Oral supplementation with antioxidants, such as tocopherol, carotenoids and polyphenols, has been proposed to protect skin against UV radiation [2,3,4]. In recent years, there has also been an increasing interest in the use of nutritional approaches, particularly those involving probiotics, to provide health benefits. Ingestion of certain *Lactobacillus* strains is thought to prevent the development of skin lesions in animal models of atopic dermatitis [5]. Furthermore, the intake of the lactic acid bacterial strain *Lactobacillus johnsonii* facilitated the early recovery of epidermal cell allostimulatory function in UV-irradiated human skin [6]. In hairless mice, probiotics could protect skin against UV-induced suppression of contact hypersensitivity, decreased epidermal Langerhans cell density and increased interleukin (IL)-10 serum levels [7]. However, the mechanism by which probiotics attenuate UV-induced skin damage remains unclear.

UV irradiation induces DNA damage predominantly by promoting the formation of cyclobutane pyrimidine dimers (CPDs). UV-induced CPDs are recognized as a molecular trigger for the initiation of immunosuppression [8]. Reduction or efficient repair of CPDs mediated by DNA repair enzymes considerably reduces the risk of photocarcinogenesis. Among the various UV-induced DNA repair mechanisms in cells, the most versatile DNA repair pathway is nucleotide excision repair (NER), which involves the xeroderma pigmentosum complementation group A (XPA) [9].

UV-induced immunosuppression is modulated by various cytokines, including IL-10 and IL-12 [10]. After skin is exposed to UV radiation, IL-10 is released from UV-stimulated keratinocytes. IL-10 is a type 2 cytokine that acts as a mediator in the induction of systemic immunosuppression following UV exposure [11,12]. Furthermore, IL-10 production and secretion by keratinocytes are triggered by UV-induced formation of CPDs [13]. Conversely, IL-12 is a type 1 cytokine, which is a major player in orchestrating both innate and acquired immune responses, and strongly induces the production of interferon-gamma (IFN-γ) from natural killer cells, leading to the development of T helper (Th) 1 responses [14]. IL-12 also suppresses UV-induced IL-10 production in keratinocytes, thereby ameliorating UV-induced immunosuppression [15]. Therefore, we speculated that milk fermented with lactic acid bacteria can exert a beneficial photoprotective effect by enhancing DNA repair through a mechanism that may involve cytokine production.

The components of milk fermented with lactic acid bacteria that contribute to its ability to protect against skin photo-damage are unclear. Many lactic acid bacteria strains used for manufacturing fermented milk products can produce exopolysaccharide (EPS), which are excreted into milk during fermentation, and prevent syneresis while ensuring desirable texture. Furthermore, recent studies showed that the EPS produced by some strains of lactic acid bacteria have immunomodulatory functions [16]. Based on these findings, we hypothesized that EPS isolated from lactic acid bacteria might have photoprotective activity. We aimed to study the mechanism by which fermented milk lessens UV-B-induced skin damage and determine the active components in milk fermented with lactic acid bacteria by evaluating erythema formation, dryness, epidermal proliferation, DNA damage and cytokine mRNA levels in hairless mouse skin exposed to acute UV-B radiation.

## 2. Results

### 2.1. Effect of Fermented Milk and Exopolysaccharide (EPS) on Skin Erythema Formation and Proliferation in Hairless Mice Following UV-B Exposure

**Figure 1:**
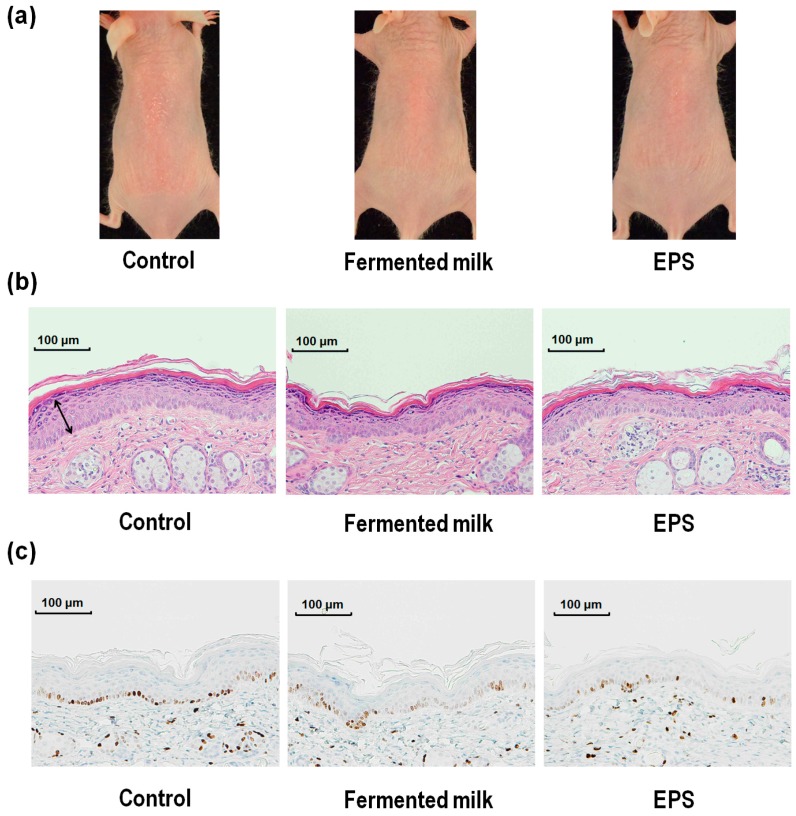
Effect of UV-B irradiation on dorsal skin from hairless mice treated with fermented milk or exopolysaccharide (EPS). The overall appearance of hairless mice exposed to UV-B irradiation (**a**); hematoxylin and eosin (H&E) stained dorsal skin sections (**b**); and Ki-67 immuno-histochemical staining of dorsal skin section (**c**) 72 h after a single dose of UV-B irradiation.

The response of dorsal skin in hairless mice to UV-B exposure was evaluated in terms of overall skin appearance, as well as hematoxylin and eosin (H&E) staining of dorsal skin sections and immunohistochemical staining for Ki-67 (Figure 1). Δa* (redness) values (a* value in UV-B irradiated skin − a* value in a non-irradiated site) were used to measure skin erythema. Skin proliferation in the different treatment groups was evaluated by measuring epidermal thickness and epidermal Ki-67 positive cell numbers.

**Figure 2:**
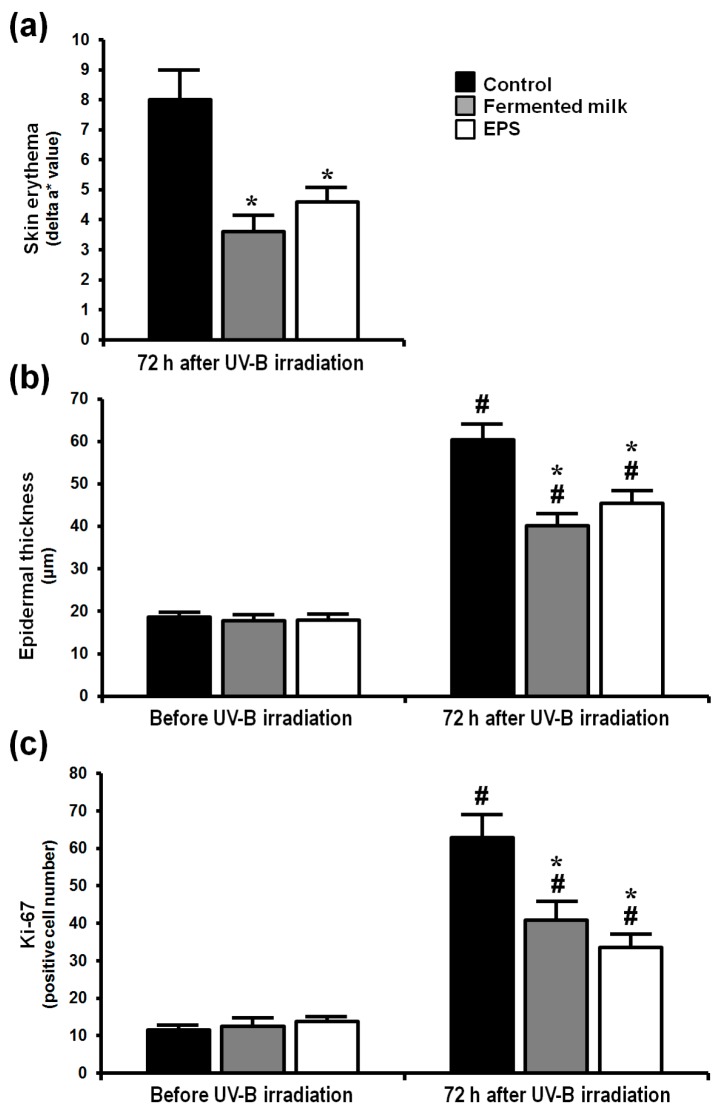
Effect of fermented milk and EPS on skin erythema and cell proliferation in hairless mice following UV-B exposure. Δa* (redness) value (a* value in UV-B irradiated skin − a* value in a non-irradiated site) (**a**); epidermal thickness (**b**); and Ki-67 positive number (**c**) were determined after a single dose of UV-B irradiation. The values are shown as means + SEM (*n* = 8). * *p* < 0.05 (vs. the control group). # *p* < 0.05 (vs. before UV-B irradiation).

At 24 h after UV-B irradiation, the Δa* values for all of the groups were similar (data not shown), but at 72 h after irradiation, the Δa* values for the fermented milk group and EPS group were significantly lower than those of the control group (Figure 2a). Epidermal thickness 72 h after UV-B irradiation was low in mice fed the fermented milk or EPS compared with those fed the control solution (Figure 2b). The number of epidermal Ki-67-positive cells 72 h after acute UV-B irradiation was also decreased in mice fed the fermented milk or EPS compared with those fed the control solution (Figure 2c).

### 2.2. Effect of Fermented Milk and EPS on Skin Barrier Function in Hairless Mice Following UV-B Exposure

**Figure 3:**
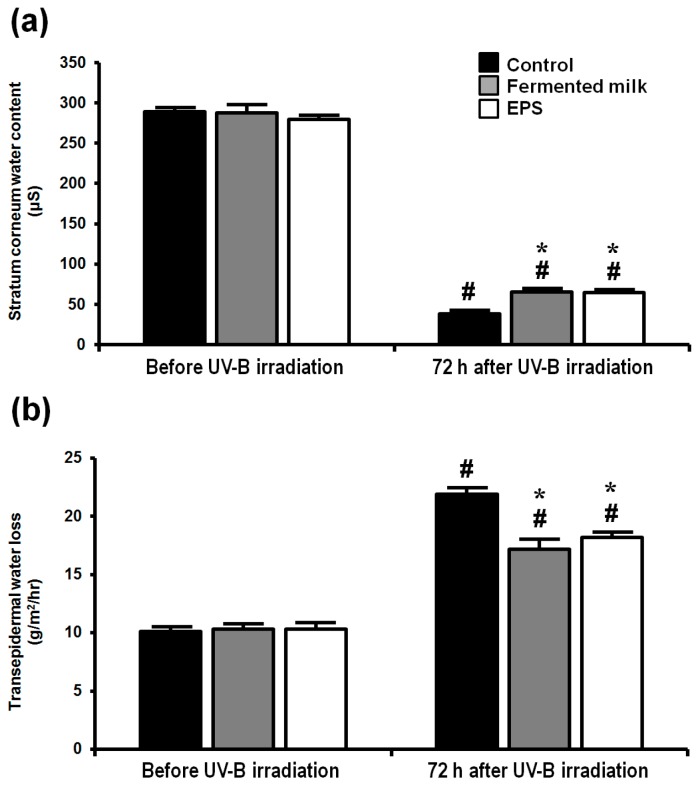
Effect of UV-B irradiation on skin barrier function of hairless mice treated with fermented milk or EPS. Stratum corneum water contents (**a**); and transepidermal water loss (**b**) were measured after a single dose of UV-B irradiation. The values are shown as means + SEM (*n* = 8). * *p* < 0.05 (vs. the control group). # *p* < 0.05 (vs. before UV-B irradiation).

Skin barrier function was assessed by determining stratum corneum (SC) water content and transepidermal water loss (TEWL) (Figure 3). The SC water contents were significantly increased in both the fermented milk and EPS group relative to the control group (Figure 3a), whereas TEWL was significantly decreased in both the fermented milk and EPS groups compared with the control group (Figure 3b).

### 2.3. Effect of Fermented Milk and EPS on DNA Damage, XPA mRNA Levels and the Ratio of Th-2/Th-1 Cytokines in Hairless Mice Exposed to UV-B

**Figure 4:**
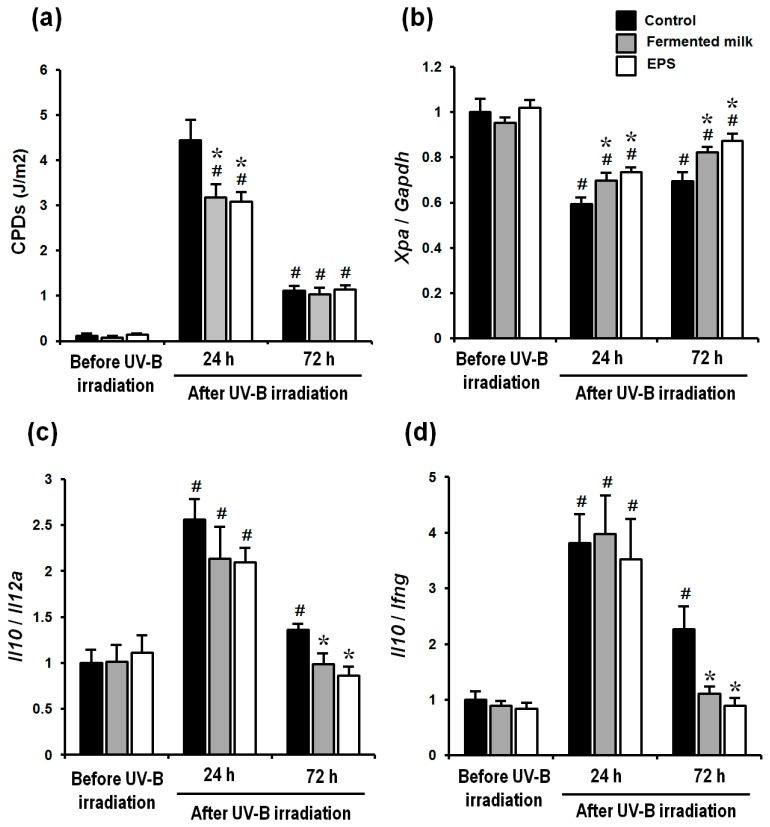
Effect of fermented milk and EPS given to hairless mice exposed to UV-B irradiation. Cyclobutane pyrimidine dimers (CPDs) (**a**); xeroderma pigmentosum complementation group A (XPA) mRNA levels (**b**); and the ratio of mRNA levels of the Th-2/Th-1 cytokines interleukin (IL)-10 /IL-12a (**c**); and IL-10/interferon-gamma (**d**) were determined after a single dose of UV-B irradiation. The values are shown as means + SEM (*n* = 8). * *p* < 0.05 (vs. the control group). # *p* < 0.05 (vs. before UV-B irradiation).

CPD contents at 24 and 72 h after UV-B irradiation were significantly increased in all groups relative to the pre-irradiation values (Figure 4a). However, mice given fermented milk or EPS showed a significant decrease in CPDs compared with control animals 24 h after UV-B irradiation (Figure 4a). UV-B irradiation significantly lowered *Xpa* mRNA levels in all groups 24 and 72 h after UV-B irradiation. However, *Xpa* mRNA levels were significantly higher 24 and 72 h after UV-B irradiation in both the fermented milk and EPS groups relative to those in the control groups (Figure 4b). Meanwhile, the ratios of *Il10*/*Il12a* and *Il10*/*Ifng* 24 h after UV-B irradiation were significantly increased in all groups, whereas the ratios of *Il10*/*Il12a* and *Il10*/*Ifng* 72 h after UV-B irradiation were significantly increased in only the control group (Figure 4c,d and Figure S1). Administration of either fermented milk or EPS significantly suppressed an increase in the ratio of *Il10*/*Il12a* and *Il10*/*Ifng* at 72 h after UV-B irradiation, compared with the control group (Figure 4c,d and Figure S1).

## 3. Discussion

This study showed that milk fermented with the lactic acid bacteria *Lactobacillus delbrueckii* subsp. *bulgaricus* OLL1247 and *Streptococcus thermophilus* 3078 significantly attenuated UV-B-induced skin damage in hairless mice, as manifested by erythema formation, skin dryness and epidermal proliferation.

UV irradiation induces DNA damage, mainly via the formation of CPDs [17]. CPDs are recognized as molecular triggers for promoting UV-induced skin damage that is characterized by immunosuppression, erythema formation and skin carcinogenesis [8]. CPD formation can be seen immediately after UV irradiation in both humans [18] and rodents [19]. Furthermore, Ueda et al. [18] showed that 24 h after UV irradiation with one minimal erythema dose (MED), an average of 60% of CPDs were removed. We therefore focused our attention on what molecules in fermented milk could affect the repair mechanisms that target CPDs formed after UV-B irradiation. Here, we showed a new protective mechanism wherein milk fermented with lactic acid bacteria suppressed CPD levels following acute irradiation with UV-B. We also showed that milk fermented with lactic acid bacteria lowered the ratio of IL-10 to IL-12a and IL-10 to IFN-γ, as well as upregulated mRNA expression of XPA, which is involved in DNA repair. UV-induced CPDs trigger the production of IL-10 [13] that in turn induces downregulation of IL-12 production followed by increased production of IFN-γ by natural killer cells [15]. Furthermore, IL-12 reduces the amount of DNA damage by stimulating NER, and this effect occurs in *Xpa* knockout mice [20]. Furthermore, the release of IL-10 by UV-irradiated keratinocytes plays an essential role in the induction of systemic immunosuppression [21,22], whereas IL-12 prevents ultraviolet B-induced local immunosuppression [23]. Thus, the ratio of IL-10 to IL-12 or IFN-γ has a crucial role in mediating the repair of UV-induced DNA damage. Therefore, administration of milk fermented with lactic acid bacteria could regulate Th-2/Th-1 cytokine responses in skin that may thereby enhance NER DNA damage repair activity and, in turn, decrease DNA damage levels. A reduction in CPDs promoted by the stimulation of DNA damage repair pathways might also contribute to attenuation of UV-induced skin damage. As it remains unclear how dietary fermented milk regulated DNA damage repair and Th-2/Th-1 response, future studies need to clarify the underlying mechanism; for example, evaluating protein levels of cytokines and other DNA repair proteins, such as xeroderma pigmentosum complementation group C (XPC) and UV-damaged DNA-binding protein.

Probiotic supplements are thought to attenuate skin damage, particularly skin inflammation, such as that promoted by UV-induced skin damage and atopic dermatitis. Supplementation with the lactic acid bacteria strain *Lactobacillus johnsonii* modulated IL-10 production in the serum and maintained Langerhans cell density at the site of UV exposure [7]. Overall, these results showed that supplementation with these probiotics could prevent the deleterious effects of UV irradiation on the skin immune system and maintain effective skin defenses against antigenic challenges. Furthermore, administration of the probiotic *Lactobacillus rhamnosus* GG can reduce the incidence of atopic dermatitis in children [24] through a mechanism that may involve an enhanced Th-1 response mediated through IFN-γ production. These findings suggest that lactic acid bacteria can modulate the immune system both at a local and a systemic level through a mechanism that involves cytokine release to affect the maintenance of skin homeostasis and to prevent skin damage. However, the active components that are responsible for the photoprotective effect of dietary fermented milk have yet to be defined conclusively.

Interestingly, we demonstrated that administration of EPS alone also had beneficial photoprotective properties that were similar to those seen for the administration of milk fermented with lactic acid bacteria. These findings suggested that the effects of fermented milk on epidermal function could be attributable to the EPS contained in the fermented milk. Several studies reported the ability of EPS synthesized by lactic acid bacteria to elicit immune responses [16]. For instance, the lactic acid bacteria strain *Lactobacillus delbrueckii* subsp. *bulgaricus* OLL1073-R1 synthesizes large amounts of EPS, which is composed of two fractions, acidic and neutral [25]. The acidic EPS fraction was shown to induce IFN-γ and IL-1α production in macrophages [26]. Moreover, acidic EPS produced by *Lactococcus lactis* subsp. *Cremoris* KSV20 from Scandinavian fermented milk could induce IFN-γ and IL-1α synthesis by mouse spleen macrophages cultivated in vitro [27]. Several other studies also showed that polysaccharides from plants can have a photoprotective effect. Indeed, oligosaccharides from aloe extracts prevented systemic suppression of T cell-mediated immune responses and suppressed the production of IL-10 by UV-irradiated murine epidermal keratinocytes both in vitro and in vivo [28]. Xyloglucan from tamarind seeds also prevents the production of immunosuppressive IL-10 in UV-irradiated skin and cultured murine keratinocytes [29]. Thus, dietary polysaccharides, including EPS produced by lactic acid bacteria, might contribute mainly to cutaneous immune regulation after UV irradiation. Further studies are needed to clarify the potential underlying mechanism for this protective effect, as is a determination of which EPS species from fermented milk could control skin immune responses to attenuate UV-B-induced skin damage.

**Figure 5:**
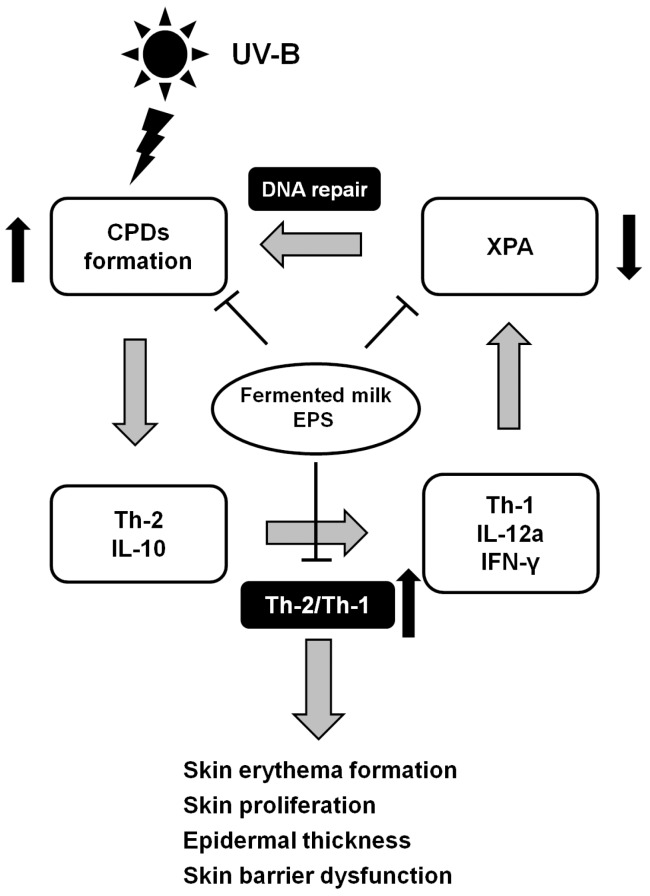
A possible scheme for the mechanism by which EPS isolated from milk fermented with lactic acid bacteria attenuated UV-B-induced skin damage in mice.

Taken together, this study showed that milk fermented with lactic acid bacteria significantly attenuated UV-B-induced skin damage that is characterized by erythema formation, dryness and epidermal proliferation in mice. Furthermore, EPS isolated from milk fermented with lactic acid bacteria attenuated UV-induced formation of DNA damage by modulating cutaneous immune balances (Figure 5). These results indicate that EPS isolated from fermented milk might contribute to enhancing the photoprotective function of the epidermis and maintaining skin health.

## 4. Materials and Methods

### 4.1. Animals

A total of 72 nine-week-old female hairless mice (Hos:HR-1, Japan SLC Inc., Shizuoka, Japan) were used in this study. All mice were housed in plastic cages (four mice/cage) in a temperature- and humidity-controlled room (24 ± 1 °C and 50% ± 10% relative humidity (RH)) under a 12-h light-dark cycle. Mice were allowed free access to the standard diet AIN-93G (Oriental Yeast Co., Ltd., Tokyo, Japan) and water. All animal experiments in this study were approved by the Meiji Co., Ltd. (Tokyo, Japan) Institutional Animal Care and Use Committee and were performed in accordance with the Guiding Principles for the Care and Use of Laboratory Animals approved by Meiji Co., Ltd. (Permit Number: 2015_3871_0264; date of approval: 14 January 2016).

### 4.2. Preparation of Samples

Fermented milk was produced using *Lactobacillus delbrueckii* subsp. *bulgaricus* OLL1247 and *Streptococcus thermophilus* 3078 (lactic acid bacteria SC-2, Meiji Co., Ltd., Tokyo, Japan) together with 10% (*w/w*) skim milk at 43 °C for 4 h (Meiji Co., Ltd., Japan). These lactic acid bacteria strains were screened for a high EPS productivity. EPS concentrations in the fermented milk were determined according to the method described by Cerning et al. [30]. Briefly, fermented milk was centrifuged at 18,500× *g* for 10 min at 4 °C and separated into supernatant and sediment fractions. EPS were precipitated by adding 2 volumes of chilled 99.5% ethanol to the supernatant, and the mixture was incubated at −25 °C overnight. The precipitate was then collected by centrifugation at 10,000 rpm at 4 °C, and the EPS concentrate was freeze-dried. The EPS concentrate contained 70 mg/1.3 g fermented milk (dry base).

### 4.3. Animal Study

After acclimatization for four days, mice were randomized into nine groups (control groups (Days 0, 1, 3), fermented milk groups (Days 0, 1, 3) and EPS groups (Days 0, 1, 3)), according to body weight. The control groups were given water at 10 mL/kg body weight. The fermented milk groups were given 1.3 g (dry base)/kg body weight of fermented milk. The EPS groups were given 70 mg/kg body weight of EPS.

Mice were given the experimental samples orally, beginning 1 week before UV-B irradiation (Day −7) and until 1 day before euthanasia. One week after beginning administration of the experimental samples (Day 0), the dorsal skin was exposed once to 20 mJ/cm^2^ emitted by a UV-B lamp (GL20SE, Sankyo Denki Co., Ltd., Tokyo, Japan, range 280–360 nm, with a peak length of ~306 nm) under isoflurane anesthesia. TEWL and SC water content were measured 7 days before and 0, 1 and 3 days after irradiation [31,32]. All mice were euthanized under isoflurane anesthesia on Days 0, 1 or 3. The dorsal skin was then quickly excised and immediately frozen at −80 °C until analysis.

### 4.4. Measurement of Transepidermal Water Loss (TEWL) and Stratum Corneum (SC) Water Content

TEWL and SC water content were assessed under standardized conditions (external temperature 24 ± 1 °C and 50% ± 10% RH) [31,32]. Both parameters were measured using a Tewameter MPA580 (Courage and Khazaka Electronic GmbH, Cologne, Germany) and SKICON 200-EX (I.B.S Co., Shizuoka, Japan) apparatus, respectively.

### 4.5. Analysis of Skin Erythema Formation

We took digital photographs of the target skin area with a color reference marker, Casmatch^®^ (Bear Medic Corp., Tokyo, Japan). Skin color was converted to the L* (lightness), a* (redness), b* (yellowness) values with Adobe Photoshop^®^ (Adobe Systems, San Jose, CA, USA) after the color compensation. Skin erythema (Δa* value) was calculated as: (a* value in UV-B irradiated skin − a* value in a non-irradiated site).

### 4.6. Histological Analysis

Dorsal skin sections were stained with H&E. Epidermis thickness (the distance from the bottom of the basal layer to the top of the granular layer) was measured with a BX-2 biomicroscope (Olympus, Tokyo, Japan) equipped with a DP-72 CCD camera (Olympus). The images were digitally assessed by image measurement and analysis using WinROOF software (Mitani Corporation, Tokyo, Japan). An average of 20 random determinations was considered to be a representative value for each individual mouse.

### 4.7. Immunohistochemistry

Expression levels of the cellular proliferation marker Ki-67 in the skin were determined. Briefly, after the deparaffinized sections were subjected to heat-induced antigen retrieval in antigen retrieval solution (citrate buffer, pH 6.0) at 121 °C for 10 min, the sections were washed with water, incubated with 3% H_2_O_2_ solution for 5 min to block endogenous peroxidase and washed three times for 5 min each with 0.01 M PBS (pH 7.4). Then, the sections were incubated with anti-rabbit Ki-67 (RM-9106-S1, Thermo Fisher Scientific Inc., Waltham, MA, USA) as a primary antibody for 50 min at room temperature and washed three times for 5 min each with PBS. To detect Ki-67 expression, the sections were further incubated with Histostar™ (Rb) for Mouse tissue (8470, Medical & Biological Laboratories Co., Ltd., Aichi, Japan) for 30 min at room temperature and washed three times with the same buffer for 5 min. Ki-67 expression in the sections was detected using 3,3′-diaminobenzidine solution as a chromogen substrate and counterstained with hematoxylin. Three different microscopic fields per plate were photographed, and Ki-67-positive cells in the skin were counted.

### 4.8. Total RNA Isolation, cDNA Synthesis and Quantitative Real-Time Reverse Transcription Polymerase Chain Reaction Analysis

Isolation of total RNA and synthesis of cDNA were performed as described previously [32]. Briefly, dorsal skin samples from each mouse were frozen in liquid nitrogen and lyophilized. Total RNA was isolated from the skin samples using a guanidine thiocyanate method [33] with TRIzol reagent (Life Technologies Corporation, Carlsbad, CA, USA) and purified with an RNeasy Mini Kit (Qiagen, Hilden, Germany). Extracted RNA was then dissolved in diethylpyrocarbonate-treated water and quantified spectrophotometrically at a wavelength of 260 nm. Reverse transcription was performed using a RivertAid First Strand cDNA Synthesis Kit (Thermo Fisher Scientific). The cDNA was stored at −80 °C prior to analysis.

Quantitative real-time PCR was performed using an ABI 7500 Fast Real-time PCR system (Applied Biosystems, Foster City, CA, USA). The respective primers and probes (TaqMan Gene Expression Assays) were designed at Applied Biosystems from gene sequences obtained from GenBank (XPA (*Xpa*): Mm00457111_m1, IL-10 (*Il10*): Mm01288386_m1, IL-12a (*Il12a*) Mm00434169_m1, IFN-γ (*Ifng*): Mm01168134_m1, glyceraldehyde-3-phosphate dehydrogenase (*Gapdh*): Mm99999915_g1). The relative expression of the gene of interest was normalized relative to *Gapdh* levels and then calculated using the 2^−ΔΔ*C*t^ method [34]. The results are expressed as arbitrary units.

### 4.9. Analysis of Cyclobutane Pyrimidine Dimers

Dorsal skin samples from each mouse were frozen in liquid nitrogen and lyophilized. DNA was isolated from the skin samples using a Qiagen QIAamp DNA Mini Kit (51304, Qiagen). CPDs were analyzed using a commercial ELISA kit (High Sensitivity CPD ELISA kit Ver.2, Cosmo Bio Co., Ltd., Tokyo, Japan). UV-C-irradiated DNA samples (Cosmo Bio Co., Ltd.), which were made by irradiating calf thymus DNA with various doses of UV-C (mainly 254 nm), including 0, 2.5, 5, 7.5 and 10 J/m^2^, were used as a standard.

### 4.10. Statistical Analysis

All data are presented as means ± standard error (SE). Data were analyzed by one-way ANOVA with post hoc analyses carried out using Dunnett’s test (time) and the Tukey test (group) (SPSS ver. 22.0, SPSS, Chicago, IL, USA). The statistical analyses of gene expression were performed at the Δ*C*_t_ stage in order to exclude potential bias due to the averaging of data transformed through the equation 2^−ΔΔ*C*t^. Differences among groups were considered to be significant at *p* < 0.05.

## 5. Conclusions

EPS isolated from milk fermented with lactic acid bacteria enhanced DNA repair mechanisms and modulated skin immunity to protect skin against UV damage.

## Supplementary Materials

Supplementary materials can be found at www.mdpi.com/1422-0067/18/1/146/s1.
