# Supplementary material for: Exopolysaccharides Isolated from Milk Fermented with Lactic Acid Bacteria Prevent Ultraviolet-Induced Skin Damage in Hairless Mice
Source: Int J Mol Sci. 2017 Jan 13;18(1):146. doi: 10.3390/ijms18010146 (PMC5297779; doi:10.3390/ijms18010146)
Supplement: Supplementary file 1 [file ijms-18-00146-s001.pdf]

Int. J. Mol. Sci. **2017**, *18*, 146; doi:10.3390/ijms18010146

Supplementary Materials: Exopolysaccharides Isolated From Milk Fermented with Lactic Acid Bacteria Prevent Ultraviolet-Induced Skin Damage in Hairless Mice

Masashi Morifuji, Masami Kitade, Tomoyuki Fukasawa, Taketo Yamaji and Masamitsu Ichihashi

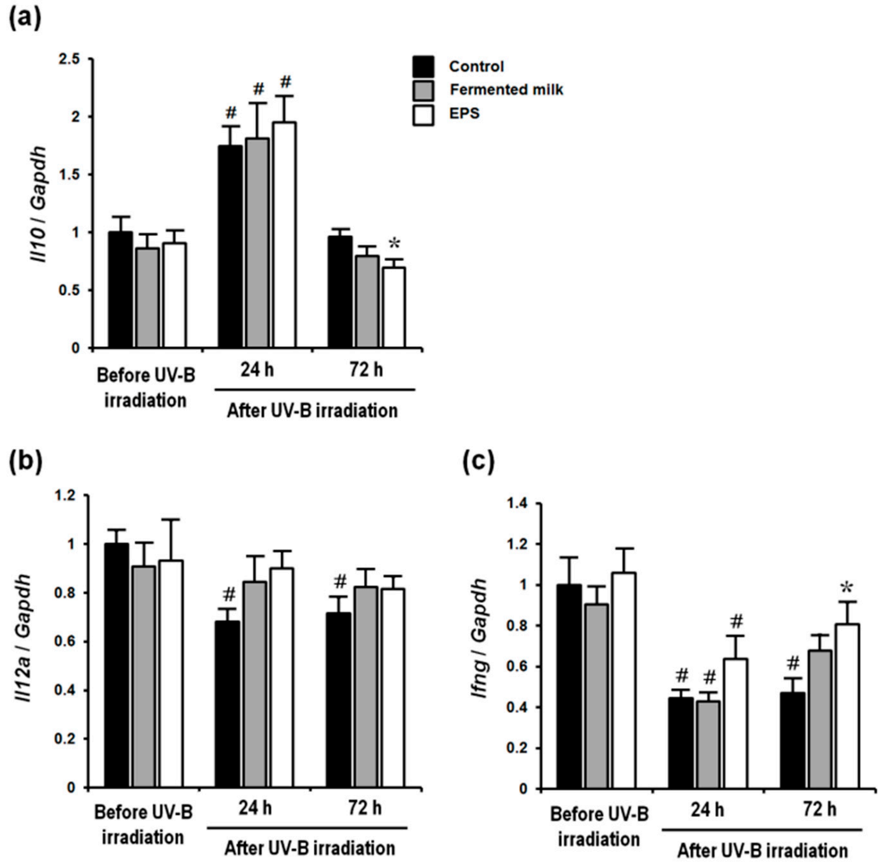

Figure S1. Effect of fermented milk and EPS given to hairless mice exposed to UV-B irradiation. Interleukin (IL)-10 mRNA levels (**a**); IL-12a mRNA levels (**b**); and interferon-γ mRNA levels (**c**) were measured after a single dose of UV-B irradiation. The values are shown as means + SEM (n = 8). * $p < 0.05$ (vs. the control group). # $p < 0.05$ (vs. before UV-B irradiation).
